# Retrospective View of the Late Improvements in Medicine and Surgery

**Published:** 1818-01

**Authors:** 


THE LONDON
### Medical and Physical Journal.

1 OF VOL. XXXIX.] JANUARY, 1818. [NO. 227.

"For many fortunate discoveries in medicine, and for the detection of numerous errors, the world is indebted to the rapid circulation of Monthly Journals; and there never existed any work to which the Faculty in EUROPE and AMERICA were under deeper obligations than to the Medical and Physical Journal of London, now forming a long, but an invaluable, series."—RUSH.

### *Retrospective View of the late Improvements in Medicine and Surgery.*

AT the commencement of our last volume, we made an apology for regarding a hint from an uncertain quarter, relative to the production of a RETROSPECT. We could now almost express our thanks for the suggestion of such an expedient, in order to get through so much business as presses upon us, and which we should be very slow in accomplishing by our ordinary mode of analysis. The number of original communications is considerable. These, indeed, usually occupy a comparatively small space; but the number of books which have gratuitously arrived, and the writers of some of which are anxious to appear before the public, embodied in our Journal, has within the last six weeks exceeded any within that period: at the same time there are several on our shelves which seem to admonish *ut incipiant in corpora velle reverti.* Among these, Dr. Robert Jackson's is perpetually reminding us of his grand reform in the treatment of fevers: whatever, therefore, relates to that subject is reserved for our analysis of his work. The present state of the epidemic fever in London will be seen by the papers in our Collectanea.

We now promise to fulfil our engagements with as much expedition as time and space will allow, and, in order to alleviate the impatience of readers and writers, offer a general outline of the state of medicine, promising to be more minute as our subsequent analyses afford opportunities.

On the subject of Physiology we shall be short. It is a field on which foreigners engage without fear, because hitherto no Hunter has appeared among them. On this account, Bichât, who in some instances approximated our English physiologist, appears on the continent as a Newton; and even Richerand and Magendie are read with patience. Of Richerand we can only say, that he has kept out of danger by offering nothing new; but, when we speak of Magendie, we confess that we lose all patience. His disgusting experiments on the stomachs of living dogs, to prove what Mr. Hunter taught us without any thing more than accurate observation, and which, having once ascertained, he disdained to prosecute by unnecessary cruelty and with unnecessary loss of time and labour, have more than once come under our notice, and have been treated with the contempt, not to say detestation, they merit.* Yet M. Magendie's paper was so well received by the National Institute, that the author not only received the thanks of that truly illustrious body, but was voted a remuneration for any expences which might attend his future experiments in prosecuting the same enquiry. This is not the only instance in which there seems on the continent an almost studied anxiety to avoid the name of Hunter. It is hardly credible to an Englishman that M. de Montegre should begin a paper on *digestion* with remarking, "Notwithstanding the numerous experiments of Reaumur, Spalanzani, Steevens, Gosse, and *other physiologists*." Why this determination to pass over a name which may be truly called "all those names in one?" Has not Mr. Hunter confirmed all that was true in the three first, corrected all their errors, and supplied what they omitted: in a word, since Mr. Hunter wrote, has any thing which can be depended upon been added to our knowledge on this important subject of digestion?

Happily our countryman is becoming more known, and, in consequence, the ignorance of others relative to his doctrines, experiments, and labours, is gradually so well

---

* See London Medical and Physical Journal, vol. xxiv. p. 525. See also Hunter's Animal Œconomy, p. 199, 2d edit.

known as to explain the cause of their backwardness in similar researches. We are not making this apology for M. Magendie, who, before he began his experiments, ought to have learned what had been done by others; still less for M. de Montegre, who, in quoting Spalanzani, could not have overlooked Hunter without a terror of his name, and a determination to avoid it. But some apology may be made for M. Cuvier's manner of receiving such communications, by the difference of his pursuits. The magnitude of his researches and of his discoveries make us less surprised, when Mr. Abernethy informs us that "M. Cuvier was ignorant of Mr. Hunter's Collection." This remark concerning *collections*, without any reference to pathological doctrines and discoveries, shows, in a lively manner, the different turn by which the enquiries of the two philosophers are directed. But, whatever we may think of continental physiology, and its application to pathology, it is impossible for us to compete with them in the accuracy with which they describe diseases; and, for the most part, it is enough if we think ourselves on a level with them in the faithfulness of their reports.

Of English Pathology we have little to say beyond what will appear when we notice Mr. Abernethy's lectures. In proportion as Mr. Hunter is better known, physiology becomes more rational, and pathology rests on a surer foundation. Spurzheim, Gordon, Phillips, and Pring, have been already noticed by us. Of the obscurity of Dr. Phillips we have often complained; we hope, however, in a work now before us, that we shall meet with better encouragement.

We cannot entirely pass over the subject of Nosology; for, however imperfect every attempt has hitherto been, it must, we conceive, by its connexion with nomenclature, radically induce a greater accuracy in our language. In the mean while, as in the artificial arrangements of the more constant productions of nature, there is reason to fear that it may have produced an inattention to that practical accuracy which is the only object of all philosophical enquiries, and, most of all, of Nosology.

It is with much satisfaction we observe the late enquiries into the Brain, Spinal Marrow, and Nerves, have produced if not a revolution of opinion, at least a recurrence to former diligence in investigating the seat of disease. We hear less of digestive organs as the only source of disorder, and of a single article of the Materia Medica as the universal remedy. It is admitted that these organs may be only sympathetically affected, and consequently that the remedy must be directed to the diseased part: that mercury may owe its celebrity to the same source as tar-water, stibium (as antimony was anciently called), and various other remedies, which, by exciting for a time a new action, induce a temporary relief. But, if the seat of the disease remains unaltered, all the symptoms return, oftentimes exasperated.

Among Nervous Diseases, Insanity, as the highest form, first presents itself. On this we have prepared long remarks, which we shall offer whenever we undertake the analysis of not less than five books on madness, now under consideration. At present, we shall only notice a new attempt at definition, as it shows that this, like all other diseases, is more readily described than defined. It is some apology for Spurzheim, as a foreigner, if he is somewhat obscure. " The incapacity of distinguishing the diseased functions of the mind, and the irresistability of our actions," he says, " constitute insanity." This might do well, if there were only one form of madness. The word *mad* is said to be derived from the Italian *matto*, to dream: but in dreaming the senses are torpid, and the eye is incapable of seeing objects before it. A reverie is called a waking dream, and delirium is called the dream of one in a fever. In what, then, do dream, delirium, reverie, and madness, differ? In all, the mind is incapable of distinguishing the suggestions of fancy from realities. In dreams, the whole voluntary muscular system is at rest, excepting only as far as respiration is concerned: in delirium, the muscles often act more powerfully than in health. In reverie, the muscles retain their power, oftentimes their actions; the senses are only torpid, because the mind is so abstracted that it disregards whatever is presented to the senses.

Delirium is a symptom of an acute disease. Sleep and reverie vanish whenever an external cause impresses any one of the organs of sense with sufficient force: on the contrary, madness is a perpetual reverie, which is so far from being removed by any powerful impulse on the senses, that the impression so excited often encreases the illusion of the mind. Thus madness is a reverie which cannot be removed by any impression on the senses, however powerful. As the patient recovers, he feels like one awaking or roused from his reverie: the impressions continue in his memory, but he is now sensible that it was all a dream.—We have entered into this long discussion in order to keep in the mind of our readers that necessity of accuracy in words, the difficulty of which we so often feel in our own composition.

It is only of late that the Spinal Marrow has attracted that attention to which its importance entitles it. Mr. Parkinson, in his ingenious little Treatise on the Shaking Palsy, has pointed out the necessity of watching carefully the state of this part, as well as of the brain, to which it is so truly an appendage that the one rarely fails to sympathise when the other is affected. Hence it is easily understood how affections of the medulla should induce epilepsy, and we are much obliged to that enlightened body, the Faculté de Médicine de Paris (analogous to our College of Physicians), for no less than eight cases in which disease in the spinal marrow, or theca, if not with certainty the cause of epilepsy, was proved, on dissection of the subjects, to be present, and the most probable cause. It is to inflammation in that part of a more chronic form that we impute those *shaking palsies* which are attended with pain.

We have received several cases of Traumatic Tetanus, but they offer nothing in contradiction of the opinion contained in our last Number, when we noticed Mr. Pring's ingenious work.

It is with pleasure we find Zootic Medicine more generally cultivated. The diseases of horses are for the most part simple and better understood; those of dogs are perhaps more important, as these animals are the closer companion of man. Mr. Blaine has written the most rational treatise on this subject: it, however, shews how much we have still to learn concerning that dreadful disease most common in the canine race, but unfortunately transferable to all others on which it has been hitherto tried. We sincerely hope Mr. B. will be encouraged to persevere in his laudable undertaking; and we recommend him, when another case occurs, not to lose it without repeated inoculations and experiments of his remedy, previous to and during the paroxysms.

The greater facility with which zootic pathology can be reduced to experimental certainty, makes us anxious to express our gratitude to those who pursue the subject on a rational system: on this account we cannot withhold a tribute of thanks to M. Teissier, who has lately been engaged in researches on the period of gestation of the females of several domestic animals. The following is a summary of the results:—

"Out of 575 cows, 21 calved between the 240th and 270th day: mean term 259½—544 between the 270th and 299th: mean term 282—10 between the 299th and 321st: mean term 303. Thus, between the shortest and the longest gestation, there is a difference of 81 days, that is, more than one-fourth of the mean duration.

"Out of 277 mares, 23 foaled between the 322d and 330th day: mean term 326—227 between the 330th and 359th: mean term 344½—28 between the 361st and 419th: mean term 390. Between the shortest and the longest gestation there was an interval of 97 days; as before, more than one-fourth of the mean duration.

"Observations were made on two she-asses only: one foaled on the 380th, and the other on the 391st day.

"Out of 912 ewes, 140 lambed between the 146th and 150th day: mean term 143—676 between the 150th and 154th: mean term 152—96 between the 154th and 161st: mean term 157½. Here the extreme interval is only 15 days, to a mean duration of 152; that is, only one-tenth.

"The mean term of seven female buffaloes was 308 days, and the extreme difference 27 days.

"The extreme gestations of 25 sows were 109 and 143 days.

"The extreme terms of gestation of 172 rabbits were 27 and 35 days; difference 8.

"In the duration of the incubation of domestic fowls, differences of from 5 to 16 days were observed. These cannot be ascribed to accidental differences of temperature; for, according to the observations of M. Geoffroi de Sainte Hilaire, the same differences are found in the duration of the development of the chickens hatched by the Egyptians in ovens.

"From the whole of his observations M. Teissier infers, that the period of gestation is extremely variable in every species. Its prolongation does not seem to depend either upon the age, or more or less robust constitution of the female, or upon the diet, the breed, the season, or the bulk of the fœtus, and still less upon the phases of the moon."

When we consider that in the inferior animals most organic actions are more regular than in man, we can now no longer be surprised at the uncertainty which sometimes attends the period of gestation in the most experienced matrons. May there not be the same variety in the growth of the fœtus as in the child before puberty?

From the time that Corvisart published his valuable Treatise on Diseases of the Heart, that organ has been more frequently suspected and found to be the seat of inflammation. It has been urged by us, when analysing Mr. Hodgson's ingenious enquiries into the effects of *inflammation* of the arteries, that the deposition of calculous matter was the general effect of that process in those vessels, and in the heart itself; yet we were little prepared to expect such a phenomenon as Dr. Weber presents us with in the Medico-Chirurgical papers of Saltzburgh. In a subject who died in his 80th year, the heart, with its pericardium and large vessels, was (to use the author's expression) completely metamorphosed.

"The petrified mass (we are told) was on the fore part full two inches in length and three in breadth, exclusive of those processes, which will be more accurately defined in describing the left side. It was about half an inch in the thickest part.

"The petrified mass on the left side, or back part, was two inches and a quarter in length, and three inches and a half in breadth, and about one inch thick. From it a process was formed over the whole posterior lower surface of the heart, of a similar petrified substance, one inch long and three inches broad, which joined the petrifaction on the right side.

The few particulars which could be learned of the patient's condition during life are as follow:—

"He possessed a stout body and lively temper; and, for the greatest part of his life, had enjoyed good health. About eleven years ago, however, he had been ill of the jaundice for about three weeks. Soon after he was seized with a violent spasm in the stomach, which had since returned almost every year till his death, most commonly towards the end of winter, and every time it lasted for eight or nine weeks. Since that period he was troubled with indigestion whenever he indulged himself a little. According to his own expression, he felt a continual throbbing sensation in the stomach, the removal of which alone would, in his opinion, make him quite well. The source of this indigestion and those spasms in the stomach, was doubtless the heart undergoing the above-mentioned metamorphosis; which, by its somewhat greater expansion, or the greater irritability of the stomach, or its heavy pressure on the latter through the diaphragm, interrupted its functions. About sixteen weeks from the time of his death, he was seized with the spasms of the stomach for the last time; and, about four weeks before his death, he is said to have suffered much for four days from a pain in his side, both day and night, which was very violent, particularly on the left side of the breast. His pulse was hard and equal, but not frequent; a violent cough, and some œdema on his arms and feet, were also observed.

"There is no doubt that this acute disorder was an inflammation of the heart, during which the consolidation of the heart with the pericardium, and that of the latter with the lungs, took place. A general dropsy succeeded; on which account the patient was received into St. John's Hospital at Saltzburg; but, when this last complaint was nearly removed, his pulse became irregular, and he died quite exhausted."

Well acquainted as we are with the facility with which the stomach adapts itself to a change of configuration, we see no reason to question that the dyspeptic symptoms arose from sympathy with the heart. That the heart is mutually affected by sympathy with the intestinal canal has been long observed; and it is with pleasure we reflect that our Baillie* should be ingenuous enough to acknowledge

* See Medical Transactions of the R. Col. of Lond. vol. iv. p. 271.

himself among those who have been deceived by such a sympathy. Corvisart, who directed so much attention to cardiac disease, fell into a similar error; but we are not certain that the French pathologist has been equally ready in acknowledging it. The following case is published by the *Societé d'Emulation*, of which Corvisart was president whilst he had the charge of Bonaparte's health.*

"A stout young man, who had retired from the army to civil life, was subject to great and frequent palpitations.—On being questioned respecting the palpitation, the patient said that it first attacked him three years before, without evident cause; had been greatly aggravated of late; but that he had submitted to no treatment, as his disease had been pronounced by all the physicians whom he had consulted, and particularly by Corvisart, to be aneurism of the heart.

"By the aid of medicine,† the man recovered his former state, but, aware of his situation, would submit to no restrictions. Corvisart was again consulted, and confirmed his former diagnosis. Another year passed without alteration, except that, for the last month, the oppression was aggravated; the cough more dry; palpitations more tumultuous; and walking impeded by a sense of painful anxiety about the præcordial region. On February 21st, 1809, after great exertion in cold weather, the patient was seized with general uneasiness, convulsion, syncope, and expectoration of florid blood. Copious vomiting of red blood and black coagula ensued; and among the food, accompanying them, were observed some gland-like and fatty substances, compact, and of an ash-white colour, so as to resemble pulmonary tubercles. They were washed, and preserved in vinegar.

"Blood continued to be discharged both by vomiting and stool, and was always accompanied with portions of the substance just described. Convulsive spasm, slight delirium, unaccompanied by

---

* One of the volumes of this Society had a portrait of Corvisart as a frontispiece, with some Latin hexameters on his having the charge of one "*Quo sospite Gallia sospes.*"

† The remedies, prescribed by Duchateau, were successive applications of leeches to the throat, anus, and thorax; antispasmodics; purgatives and laxative injections; chicken broth mixed with orange-flower water, and syrup of violets; and lemonade.

severe suffering, weakness and concentration of the pulse, extinction of the voice, insatiable thirst, and dryness of the tongue, were followed by death, which took place on the fourth day.

*Dissection on the 28th of February.*—All the thoracic organs were sound; as were the stomach, liver, gall-bladder, spleen, and pancreas. On opening the intestinal canal from the pylorus, its duodenal and jejunal portions appeared healthy; but the ileum, for about two feet from its junction with the colon, was livid and gangrenous throughout. The colon itself was distended and blackish; its whole right portion sphacelated, and perforated to the extent of a crown-piece. The membranes were so much altered as to break beneath the finger. The whole adherent portion of omentum, which had escaped destruction, was of a dark-grey colour; the detached part had escaped into the intestine, and had followed the route of the fluids evacuated by stool and vomiting. The intestine, opened throughout, presented internally the same black colour; the mucous membrane loose, in consequence of gangrene."

These difficulties should impress us with mutual candour for the sake of each other, and for the sake of our patients, with a due attention to all cases, however palpable they may seem. There is reason to believe that Mr. Hunter, to whom we owe so much for fixing many of the laws of sympathy, was in this respect mistaken in the seat of his own disease. By the account of some cases, which will be seen in our Analysis of the Medico-Chirurgical Transactions, we can hardly believe that inflammation in the stomach, so violent as to produce the symptoms described by Mr. Hunter in his Treatise on the Blood, and afterwards by Sir Everard Home, in his Life of that eminent physiologist, could have ended otherwise than in suppuration of that important organ, and, in consequence, death. Yet, in the examination of Mr. Hunter's body, the stomach was found entire, and part of the heart and larger arteries ossified.

A striking proof how inferior the French remain in pathology, notwithstanding all their advantages from morbid anatomy, occurs in a case described by Dr. Brichteau. The subject died suddenly, and was examined in the Hotel Dieu. The disease proved aneurism of the aorta, in which the pressure of the aneurismal sac had produced an absorption of part of the œsophagus and of the vertebræ, excepting, as a was long since remarked by Dr. W. Hunter, that the cartilages remained entire. That the aneurism should open into the œsophagus is certainly a rare case. This may easily be understood, because the yielding nature of that tube would, for want of resistance, make it less liable to absorption. That the coagulum which forms the sac induces absorption wherever it presses, and lastly is absorbed itself, is well known. But the paper, part of which we shall transcribe, is chiefly curious on account of the apparently studied caution with which every English pathologist is kept out of sight.

"That the aneurism, just described, must have existed for some time is evident from the organic changes which its parietes had undergone, from the condition of the vertebræ, and from the singular aspect and configuration of the aperture of communication between the sac and the œsophagus. The orifice was not ruptured; and the inequalities which it presented, as well as other parts of its circumference, were smooth and well organized. The irruption of blood into the œsophagus had been prevented by the membranous and solid coagula, and the circulation carried on in this adventitious cavity, which represented a portion of the aorta. In proportion as the sac, adherent to the œsophagus, was *worn away by the friction*, coagula were probably deposited there, which consequently acquired adhesions to the surrounding parts, and served to prevent the escape of the blood into the œsophagus, until by *some unknown cause* the feeble barrier was broken down, which the lateral effort of the blood had not been able to destroy. Verbrugge asserts, that a similar obstacle long preserved the life of an individual suffering from aneurism, which communicated with the trachea. Every thing leads us to infer, that, in this case, the disease was obscurely marked. M. Husson, a disciple of Corvisart, well versed in the diagnosis of lesions of the heart and large vessels, examined the patient with great attention only twelve hours before death, and did not suspect aneurism. This obscurity seems to depend on the situation of the disease, deeply developed between the vertebral column and the heart, and on the accidental disposition of the aneurismal sac. By these two peculiarities, the affection was rendered less severe in its consequences, and more difficult of detection by its symptoms. The caries of the bones, so remarkable in this case, was the result of reiterated *friction*, caused by the pulsations of the aneurismal tumour. It were useless, I think, to refute the opinion of those who have asserted that this phenomenon is produced by the action of the fluids contained in the aneurismal sac; since the cartilages, in this case, suffered no change, owing to their elastic property. Corvisart has remarked, that moveable bones, such as the clavicle, are frequently displaced by the pulsations of the tumour. The excavation of bones, resulting from aneurism, are attributed by Scarpa to the action of the lymphatic system, which eats away the compressed parts, destitute of sufficient vitality whereby to resist its influence. He compares the ravages, thus exercised by the lymphatic vessels, to the devastation of worms on substances attacked by them. It sometimes happens that the parietes of the sac wear away the contiguous bones, without themselves suffering extenuation. Verbrugge ascribes this phenomenon to the activity of nutrition which exists in these parts, and rapidly repairs the losses which they have sustained."

Could one believe that, sixty years ago, Dr. W. Hunter had described exactly this progress in aneurism, and the same appearances in the vertebræ, and had assigned a most rational final cause for the power of the cartilages to preserve themselves;* and that Scarpa, who came to London in 1782, to learn the mode of examining the lymphatic system, describes in the above passage nothing more than the "*worm-eaten caries*" of Mr. J. Hunter?

Among the other extraordinary organic phenomena, few will be found more so than a case in the Repository, in which sixteen measured pints of dark coloured and offensive urine were at one time drawn by the catheter from the urethra of a woman, 45 years of age, and ten days after her delivery. The case presents nothing else remarkable but the recovery of the patient after such a quantity of fluid had been lodged in the bladder, ureters, and probably the pelvis of the kidneys.

In a former Number we mentioned some cases in Germany, in which the substance of the uterus had been freely cut into, and a part taken away, without any immediate ill consequence. One of our contemporary Journals now gives a case in which the whole was removed. There is, however, a similar one in a collection to which we cannot immediately

---

* Medical Observ. and Inquiries, vol. i. p. 348; Lond. 1758.

turn in which a mad woman amputated the whole of the uterus, like the late case, in the condition of procedentia. In the present instance, the removal was by ligature. As there is a period of life when this viscus is useless, at a time too when it is most liable to disease, we conceive these cases and the possibility of such an operation ought to be kept in view. May not a procidentia be gradually or artificially induced, or may not the operation in some cases be attempted with the uterus *in situ*?

The contraction of the uterus has produced the protrusion of a child thirty-six hours after the mother ceased to breathe. In this there is nothing astonishing to those who make a proper distinction between absolute death, and such a cessation of the functions necessary to support life as can never be restored. Mr. Ch. Clarke relates something of this kind in a volume of the Medico-Chir. Transac.; and Mr. Hunter remarked the contraction of the injected vessels in the placenta of a cow, forty-eight hours after its separation from the body of the animal. But we cannot help remarking, that, in all cases of death at an advanced period of gestation, the Cæsarian operation should be instantly performed, if the child cannot be otherwise extracted with safety.

Some useful papers have lately appeared on Phlegmasia Dolens. We very much wish our correspondents would examine the analogy between this disease and the Barbadoes leg, or *morbus glandularis* of the inter-tropical regions. We prefer this term as sanctioned by Dr. Caddell in his ingenious inaugural dissertation. The word Elephantiasis is now, by common consent, applied to the disease so named by Aretæus, whose description, though inaccurate in those parts which are not exposed to view, is so perfect in other respects, that it is impossible to question its true character.

Morbid Poisons begin to be better understood, though the term is not so much in use as it ought to be. For want of such a natural division of disease, we have long histories of cases and symptoms without that order of which Celsus, though unacquainted with the subject, has left us an invaluable example, and which Mr. Hunter, unacquainted with the language of Celsus, has taught us how to improve. Is this an additional proof that the premature attempt at artificial arrangement has proved injurious to pathology, by directing our attention more to definition, in subjects in which the *certi fines* are ascertained with so much difficulty, instead of teaching us that minute description which ought to precede every attempt at arrangement? It is not easy to ascertain the laws of contagion, but, where similar local affections seize a number of individuals similarly situated or circumstanced, such an affection must at least have one common cause, whether by communication or not. The hospital gangrene has been well understood since Dr. Rollo detected and described it in the Infirmary of the Royal Arsenal at Woolwich, and Dr. Trotter and other naval physicians in the fleet. In a late Number we had occasion to remark a peculiar and most formidable disease which was discovered in the pudenda of several females much under the age of puberty, and in whom there could be no suspicion of any improper connexions. In the Bulletin of the last year from the Faculté of Medicine at Paris, we find a paper by Dr. Baron, on a gangrenous affection of the mouth peculiar to very young subjects. The author describes his cases with the accuracy we always expect to meet with in the French and other continental physicians, but his pathological reasoning affords a further proof of the want of that closeness which Mr. Hunter has introduced into the London school. For want of this, the disease is alternately imputed to scrofula, scurvy, and other causes. The truth is, that the whole is imputable to one general cause, the same as that which induces hospital fever or gangrene, and of which we had so striking an instance in our own Foundling Hospital, when that building was over crouded, and the inhabitants fed with more caution than real prudence. The disease once ascertained, the remedy is not less simple, though not always accessible, namely, a more free ventilation, a more generous diet, and, most of all, a change of residence.

We were much in hopes, since the more *enragés* zealots have been silenced in their opposition to Cow-Pox, that this invaluable discovery would be left to its own merits, and continue to gain in public estimation, as it has progressively done since its first discovery. But this most desirable suspension of hostilities has been strangely broken in upon by one who, of all others, should have been best satisfied with the general pacification. It was with great concern, that we found in Mr. Moore a renewal of animosities almost forgotten. Of his "History of Vaccination," we shall be obliged to say something, but it will be as little as we decently can, and this entirely from a wish, that the good cause may continue uninterruptedly progressive.

The facility with which the bodies of the defunct are procured in Paris, gives the French a great advantage in Morbid Anatomy. The ninth volume of the Dict. de Science Medic. (a work we are sorry to see not kept up with the spirit with which it commenced) contains, under the article Diaphragm, an ingenious paper which, like many others from the same source, is only objectionable on account of its length. The foreign writers, in imitation of Vanswieten, seem determined to exhaust every subject they touch. That the diaphragm has been wounded, ruptured, suppurated: that, even from original conformation, the contents of the abdomen have passed into the thorax through too great an aperture at the natural perforation, has been long known. It is true, sufficient attention has not at all times been paid to it. We recollect Mr. Astley Cooper, when a young professor at Surgeon's Hall as it was then called, related the case of a parish female pauper, who had through life been accused of laziness. No disease could be discovered, but, whenever the least degree of labour was required, she became ill. Her disposition was so good, that she in vain undertook exertions inconsistent with her original formation, and, in one of these exertions, expired! On examination, the original perforation of the diaphragm was found so patent that the least over-exertion of the abdominal muscles would force the contents of that cavity into the thorax. Hence arose an immediate difficulty of breathing, with long-continued nausea and constipation. The ingenious professor remarked, that race-horses, when preternaturally animated, not uncommonly fell suddenly from a rupture of the diaphragm. Was it to this that Horace alludes, when he speaks of a horse too much urged after passing his prime.

"Solve senescentem maturè sanus equum, ne

Peccet in extremum, ridendus, et *ilia ducat.*"

Whether Scarpa, to whose exertions we owe so much, has noticed this form of rupture in his treatise, we have hitherto not yet had leisure to examine.

Among the attempts at improving practical surgery, we are sorry to see the revival of the operation of perforating the tympanum. We have witnessed the ill effects of it in several instances. Cheselden, who some years ago proposed it in the Gentleman's Magazine, abandoned it long before his death. Mr. A. Cooper, who revived it, we have reason to believe, now discourages it, and we can, from our knowledge, assert, that it is frequently useless, and not always unattended with danger. We are informed, that Mr. Wright, of Bristol, advocates this operation, but have not hitherto had time to examine his experiments. Mr. Carpue has, with much greater success, revived the Taliacotian operation for restoring the nose. Such has been his success, that several other sufferers, from various causes, have applied to him, and in most, if not all, of those which he has undertaken, he has been uniformly fortunate.

We have always regretted the time which has been so misspent in the attempts at Animal and Vegetable Chemistry. In the former, nothing has been done which lessens the accuracy of J. Hunter, though probably he knew but little more of philosophic chemistry than of Greek. In vegetables, we have had various analyses, the substance of which we almost forget before we arrive at the end. There is, however, one important part of animal chemistry which was obscurely treated by Austin, afterwards by Wollaston, with an accuracy characteristic of the man, but on specimens not sufficiently numerous. We are glad to find, that Dr. Marcet has taken up the subject. Not only his chemical knowledge, but his large opportunities during a length of hospital-duties unknown to Wollaston, and only begun by Austin, give us hopes of deriving information equal to all these advantages.

We are almost afraid to touch on one division of our duties. Dr. Bancroft has published an appendix to his heavy book on Yellow Fever. The introduction to this Appendix, or Sequel as it is entituled, consists of twenty pages, and the sequel itself of 476, closely printed in 8vo. We dread to look for the Hankey! Indeed, we enter this necessary part of our duty with much reluctance, and trust our readers will have some mercy on us whilst we wish to spare them the puzzle arising from terms ill defined, facts differently stated, and diseases arising from various causes. How can we be expected to unravel such a knot; but to cut it would be still more unphilosophical than to acknowledge our incapacity.

We have already hinted at the number of publications with which we are honoured by their authors or publishers. We cannot fail to particularize one with much satisfaction—"The Dublin Hospital Reports and Communications in Medicine and Surgery." The volume contains not less than seventeen papers of different value, as may be supposed; but a collection from such a source cannot but prove an useful addition to our well-authenticated records. We are glad to see some long papers on fever from a quarter in which we may expect diseases similar to our own, and in which the state of the country has proved an infinitely more prolific source of fever among all classes.

---

*For the London Medical and Physical Journal.*

